# Copper Ion Attenuated the Antiproliferative Activity of Di-2-pyridylhydrazone Dithiocarbamate Derivative; However, There Was a Lack of Correlation between ROS Generation and Antiproliferative Activity

**DOI:** 10.3390/molecules21081088

**Published:** 2016-08-20

**Authors:** Tingting Wang, Yun Fu, Tengfei Huang, Youxun Liu, Meihao Wu, Yanbin Yuan, Shaoshan Li, Changzheng Li

**Affiliations:** 1 Department of Molecular Biology & Biochemistry, Xinxiang Medical University, Xinxiang 453003, Henan, China; wangting2855@sina.com (T.W.); fuyun9801@163.com (Y.F.); htengfei@yahoo.com (T.H.); liuyouxun@126.com (Y.L.); meihaowu1995@163.com (M.W.); 2 Henan Collaborative Innovation Center of Molecular Diagnostics and Laboratory Medicine, Xinxiang Medical University, Xinxiang 453003, Henan, China; 3 Department of Surgery, The Third Affiliated Hospital of Xinxiang Medical University, Xinxiang 453003, Henan, China; yuanyanbingo@yahoo.com

**Keywords:** di-2-pyridylhydrazone dithiocarbamate *S*-propionic acid, antiproliferative activity, apoptosis, autophagy, cell cycle arrest, antagonistic effect, copper complex, ROS-uncorrelated cytotoxicity, tumor microenvironment, antitumor mechanism

## Abstract

The use of chelators for cancer treatment has been an alternative option. Dithiocarbamates have recently attracted considerable attention owning to their diverse biological activities; thus, the preparation of new dithiocarbamate derivatives with improved antitumor activity and selectivity as well as probing the underlying molecular mechanism are required. In this study, di-2-pyridylhydrazone dithiocarbamate *S*-propionic acid (DpdtpA) and its copper complex were prepared and characterized, and its antiproliferative activity was evaluated. The proliferation inhibition assay showed that DpdtpA exhibited excellent antiproliferative effect in hepatocellular carcinoma (IC_50_ = 1.3 ± 0.3 μM for HepG2, and 2.5 ± 0.6 μM for Bel-7402). However, in the presence of copper ion, the antiproliferative activity of DpdtpA was dramatically attenuated (20–30 fold) owing to the formation of copper chelate. A preliminarily mechanistic study revealed that reactive oxygen species (ROS) generation mediated the antiproliferative activity of DpdtpA, and accordingly induced apoptosis, DNA cleavage, and autophagy. Surprisingly, the cytotoxicity of DpdtpA copper complex (DpdtpA–Cu) was also involved in ROS generation; however, a paradoxical relation between cellular ROS level and cytotoxicity was observed. Further investigation indicated that DpdtpA could induce cell cycle arrest at the S phase; however, DpdtpA–Cu lacked this effect, which explained the difference in their antiproliferative activity.

## 1. Introduction

Hepatocellular carcinoma (HCC) is the third leading cause of cancer-related deaths worldwide, and the incidence of HCC is the highest in Asia and Africa. Certain patients may benefit from resection, though mostly transiently. Tumor metastasis is responsible for approximately 90% of all cancer-related deaths [1]. It has been recognized that a metastatic tumor stems from a primary tumor that acquires an additional ability to invade the extracellular matrix (ECM). In the early expansion of primary tumors, tumor cells secrete signaling molecules to recruit myeloid cells that provide a platform of chemokines, growth factors, and matrix-degrading enzymes [2], which constitute the tumor microenvironment. It has been demonstrated that tumor microenvironment plays an important role in the metastasis process [1,3]; therefore, targeting the tumor microenvironment for cancer therapy is an interesting topic for medicinal research [4].

Trace metal elements, such as copper, iron, or zinc, are essential for most organisms for cell growth. The metal ions are present in the biological system, either in the metalloproteins or in metal-labile pools. Cytochrome oxidase, zinc-copper superoxide dismutase, lysyl oxidase, and several transcription factors require copper for activity [5]. Increasing data also showed that low levels of copper directly result in inhibition of angiogenesis and cancer cell growth, which is dependent on angioproliferation [6,7]. It is well known that cancer cells have an increased demand for iron and copper to maintain robust cell proliferation and metastasis; thus, the use of chelators for cancer treatment has been an alternative option.

Dithiocarbamates constitute a group of sulfur-containing compounds with a strong chelating ability toward metal ions [8], which can modulate the key proteins involved in biological processes, such as apoptosis, oxidative stress, transcription, and degradation of proteins [9]. This aroused an interest to explore their potent applications both as anticancer agents and for treatment of many other disease conditions [10,11,12,13]. Owing to their versatile biological activities, many novel dithiocarbamate derivatives have been synthesized and their anticancer activities have been evaluated [14,15,16,17,18]. Moreover, many metal complexes, such as cisplatin, exhibited a significant antiproliferative activity, and it is known that dithiocarbamates exhibit a marked affinity to metal ions, especially transition metals. Thus, many complexes of dithiocarbamates have been prepared and investigated for their antitumor activity. Some of them, such as gold, platinum, and palladium complexes, showed a prospective use in cancer therapy and were eligible for preclinical observation [17,18,19,20]. Preliminary mechanistic studies revealed that dithiocarbamate derivatives could act as nuclear factor kappa B (NF-κB) inhibitors [21], proteasome inhibitors [18], DNA intercalators [22], and inactivators of numerous metal-containing enzymes [23]; however, the detailed underlying molecular mechanisms are still largely unknown.

We aimed to target the molecules whose functions depend on the transition metals in the tumor microenvironment, such as matrix metalloproteinases, lysyl oxidase, and vascular endothelial growth factor, which can be used as drug targets to inhibit tumor growth or metastasis. As mentioned above, dithiocarbamates’ excellent biological activities correlated, at least partly, with their great affinity to metal ions; however, a very strong affinity toward transition metals may also bring undesirable consequences, such as direct inactivation of enzymes required for cell growth. Neutralization of their affinity to metals by introduction of heterozygous coordination atoms into the structural unit may be a good option. In this study, we introduced 2-dipyridylketone, which has nitrogen coordination atoms, into the dithiocarbamate system, to develop a novel hydrazone-derived dithiocarbamate. Next, the novel di-2-pyridylhydrazone dithiocarbamate derivative was preliminarily evaluated for its antitumor activity against hepatocellular carcinoma, and showed excellent antitumor activity. The preliminary mechanistic study revealed that the new dithiocarbamate derivative could induce reactive oxygen species (ROS) generation, cell cycle arrest, apoptosis, and autophagy. To evaluate its copper-chelating ability that can be potently used to disturb the tumor microenvironment when administered intravenously, the antiproliferative activity of the dithiocarbamate derivative in the presence of copper chloride was also evaluated. Surprisingly, the addition of copper chloride significantly decreased the antiproliferative activity of the dithiocarbamate derivative and was further attributed to the formation of copper complex. Interestingly, it is the first study to find that there was no correlation between the antiproliferative activity and ROS generation by the dithiocarbamate copper complex. Besides, the attenuated antiproliferative activity of the new dithiocarbamate derivative upon chelation with copper ion could be favorable in the animal model studies owing to the lower cytotoxicity to other cells, both in the normal tissues and in the tumor microenvironment.

## 2. Results

### 2.1. Antiproliferative Effect of DpdtpA

**Figure 1:**
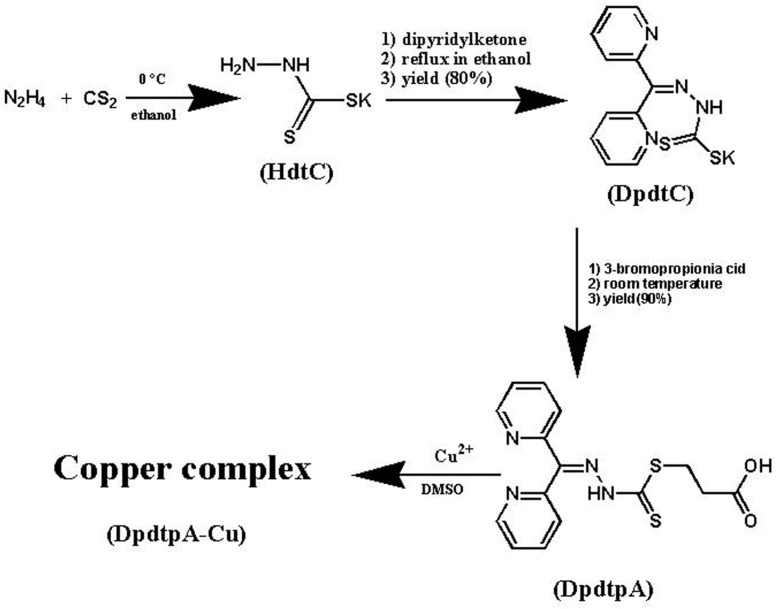
The synthetic rout of DpdtpA and its copper complex.

Dipyridylhydrazone dithiocarbamate *S*-propionic acid (DpdtpA) was prepared by a three-step reaction as described in Figure 1. First, hydrazine was reacted with carbon disulfide to form hydrazine dithiocarbamate (HdtC); after the addition of dipyridylketone, dipyridylhydrazone dithiocarbamate (DpdtC) was prepared. DpdtpA was prepared by the reaction of DpdtC with 3-bromopropionic acid. DpdtpA copper complex was obtained by mixing the DpdtpA solution with copper chloride. The new compounds, except the non-isolated intermediates of each step, were traced by TLC, and purified by flash chromatography. Finally, the chemical structures of the new compounds were determined by NMR, IR, and HRMS spectra. HPLC and NMR showed that the compounds have adequate purity (>95%, see Supplementary Materials), indicating that the compounds can be used for the biological assay.

**Figure 2:**
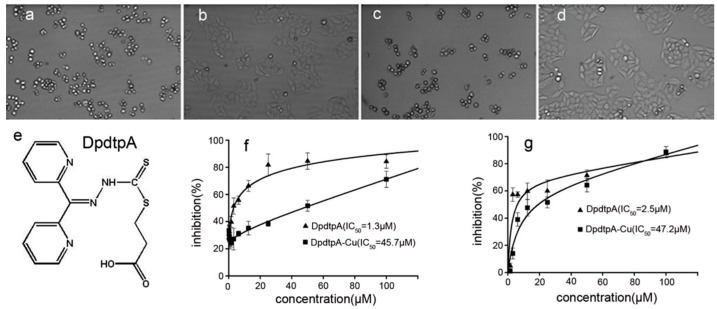
The chemical structures of DpdtpA and the proliferation inhibition assay. The effect of DpdtpA and DpdtpA–Cu on the morphology of HepG2 cells: (**a**) 5 μM DpdtpA after 16-h treatment; (**b**) 5 μM DpdtpA–Cu after 16-h treatment; (**c**) 2.5 μM DpdtpA after 48-h treatment; (**d**) 2.5 μM DpdtpA after 48-h treatment; (**e**) structure of DpdtpA; (**f**) proliferation inhibition by DpdtpA or its copper complex in Bel-7402 cells, IC_50_ = 1.3 ± 0.3 μM for DpdtpA, 45.7 ± 3.6 μM for its copper complex, respectively; and (**g**) proliferation inhibition by DpdtpA or its copper complex in HepG2 cells, IC_50_ = 2.5 ± 0.6 μM for DpdtpA, 47.2 ± 4.1 μM for its copper complex, respectively.

Next, we screened the cytotoxicity of DpdtpA against hepatocellular carcinoma (Figure 2e). The dose–response curves are depicted in Figure 2. As shown in Figure 2f,g, DpdtpA caused significant inhibition of the growth of HepG2 and Bel-7402 cells (IC_50_ < 3 μM); however, the difference in maximal inhibition of both cell types was obvious. For HepG2 cells, the inhibition percentage was only about 60% at 50 μM (Figure 2g), whereas the inhibition percentage was higher for Bel-7402 cells at the same concentration of DpdtpA (Figure 2f). In view of the critical physiological functions of copper, such as vascularization, and the excellent copper-chelating ability of DpdtpA, the inhibition of proliferation by DpdtpA was also investigated in the presence of copper ion. Unexpectedly, the growth inhibition dramatically decreased for both cell lines, and approximately a 20- to 30-fold decrease in the proliferation inhibition was observed based on the IC_50_ value compared to that of DpdtpA. For a detailed explanation, a morphological analysis was conducted. As shown in Figure 2a–d, DpdtpA caused the cells to be round; however, the addition of copper ion significantly attenuated this action, showing a protective effect or an “antagonistic effect” that is rarely reported.

### 2.2. New Species Determined by the Spectral Study

**Figure 3:**
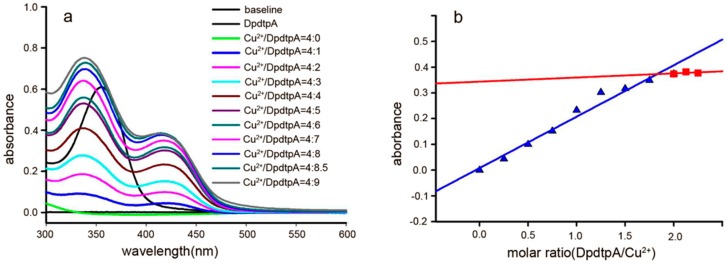
UV-visible spectra of DpdtpA copper complex and the relation between the absorbance and the molar ratio. (**a**) Spectra of CuCl_2_ alone and in the presence of various concentrations of DpdtpA; the molar ratio is indicated in the figure; (**b**) Plot of the absorbance of the copper complex at 420 nm vs. the molar ratio of DpdtpA:Cu^2+^, a 2:1 ratio. The red line was regressive line of molar ration below 2 and the blue was regressive line of molar ration over 2.

The “antagonistic effect” of copper ion on the antitumor activity motivated us to reveal the underlying detail. We speculated that the “antagonistic effect” could stem from a newly formed species. To support this hypothesis, the absorption spectra of various concentrations of DpdtpA in Tris-HCl buffer (pH 7.2) at a fixed copper concentration were obtained as previously reported [24]. As shown in Figure 3a, the copper(II) solution had no absorption in the investigated range of wavelengths (300–500 nm); after addition of DpdtpA to the copper solution, a new absorption reading appeared at approximately 420 nm, which was different from the absorption of DpdtpA (approximately at 353 nm). The bathochromic shift indicated the formation of a new species due to a reaction between DpdtpA and copper ion. For further determination of the composition of the new species (and the molar ratio of DpdtpA:Cu), various amounts of DpdtpA were added to the copper chloride solution. The new peak gradually increased until the molar ratio of DpdtpA:Cu = 2. The absorbance (at 420 nm) at each addition vs. the molar ratio of DpdtpA:Cu^2+^ was plotted (Figure 3b). A 1:2 molar ratio of Cu^2+^/DpdtpA was determined. The DpdtpA copper complex was further prepared in ethanol based on this molar ratio, and characterized by IR. The structure of the complex was tentatively proposed as shown in Figure S5.

### 2.3. DpdtpA and Its Copper Complex (DpdtpA–Cu) Induced ROS Generation

**Figure 4:**
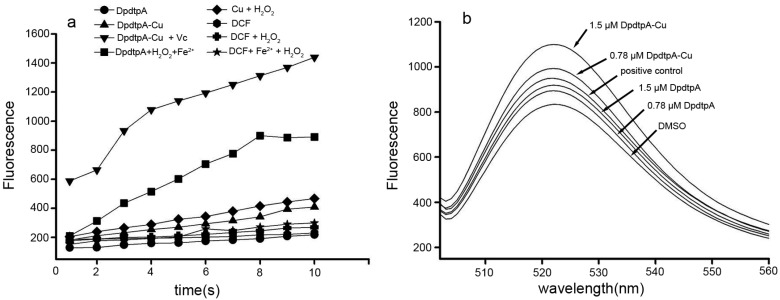
DpdtpA and its copper complex induced ROS production in vitro and in vivo. (**a**) In vitro ROS generation by iron or copper via a Fenton-like reaction; the ROS content was measured by DCF fluorescence; (**b**) In vivo, the ROS production after exposure of DpdtpA and its copper complex. DMSO was used as a control.

It is a prevailingly accepted concept that the antiproliferative activity (or cytotoxicity) of many drugs stems from their ability to generate ROS. DpdtpA may chelate iron except copper, thus it is involved in Fenton-like reaction. The assessment of ROS production in vitro is shown in Figure 4a, the fluorescence intensities of dichlorofluorescein (DCF) in the presence of DpdtpA were higher than those in the absence of DpdtpA, indicating that iron DpdtpA complex produced more ROS via a Fenton-like reaction. In contrast, DpdtpA–Cu has a weaker ability to generate ROS; however, in the reduced environment (in the presence of ascorbic acid (Vc)), DpdtpA–Cu resulted in the highest production of ROS. To further confirm these results, an in vitro study was carried out to investigate the drug-induced intracellular ROS production. As shown in Figure 4b, both DpdtpA and its copper complex induced ROS generation in a concentration-dependent manner. The fluorescence intensities of DCF in copper complex-treated cells were significantly greater than those of DpdtpA, which might be related to the redox feature of Cu^2+/1+^ complexes, because the copper(I) complex could also react with an oxygen molecule to form the superoxide radical except with H_2_O_2_ [25].

### 2.4. Cellular DNA Fragmentation by DpdtpA and Its Copper Complex

**Figure 5:**
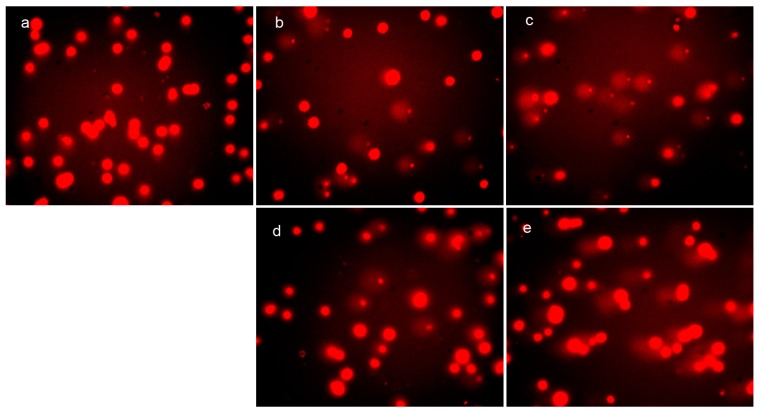
In vivo ROS generation and DNA fragmentation (comet tail) induced by DpdtpA and its copper complex. Comet tail: (**a**) control; (**b**) 0.78 μM DpdtpA; (**c**) 1.56 μM DpdtpA; (**d**) 0.78 μM DpdtpA–Cu; and (**e**) 1.56 μM DpdtpA–Cu.

It has been demonstrated that ROS cause oxidative damage of DNA and proteins, which correlates with the cytotoxicity. To investigate the correlation between ROS generation and DNA fragmentation, the comet tail induced by DpdtpA and its copper complex was measured. As shown in Figure 5, the abundance of the comet tail or DNA damage in the presence of DpdtpA (or DpdtpA–Cu) was higher than that of the control and was concentration-dependent (Figure 5b–e). However, there was no obvious difference between the DpdtpA- and DpdtpA–Cu-treated groups. Generally, DNA damage is directly related to the extent of ROS production; however, this correlation was not evident in our in vitro and in vivo studies, at least for DpdtpA–Cu.

### 2.5. DpdtpA and Its Copper Complex Induced Cellular Apoptosis

**Figure 6:**
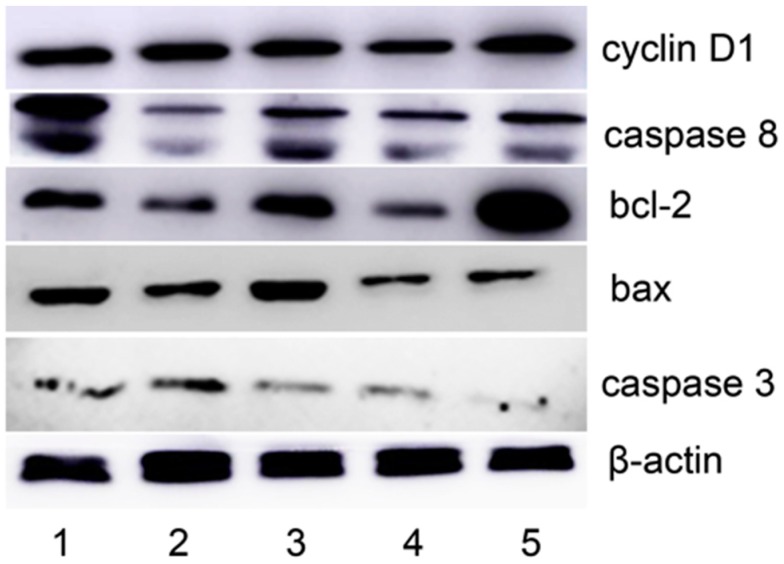
Western blotting analysis of the changes of apoptosis-related genes: 1, 1.5 μM DpdtpA; 2, 3.12 μM DpdtpA–Cu; 3, 1.56 μM DpdtpA; 4, 3.12 μM DpdtpA; and 5, DMSO control.

ROS as a messenger that plays a crucial role in the cell growth, and excessive ROS production induces apoptosis. The previous experimental results implied that apoptosis might be involved. To support this hypothesis, apoptosis-related proteins were monitored by Western blotting. As shown in Figure 6, upregulation of caspase-3, caspase-8, and bax was observed; however, bcl-2 was downregulated. This supported that an apoptotic process was involved, and indicated that both DpdtpA and DpdtpA–Cu shared similar mechanisms; however, the weaker growth inhibitory effect of DpdtpA–Cu was not explained.

### 2.6. The Effect of DpdtpA and Its Copper Complex on the Cell Cycle

**Figure 7:**
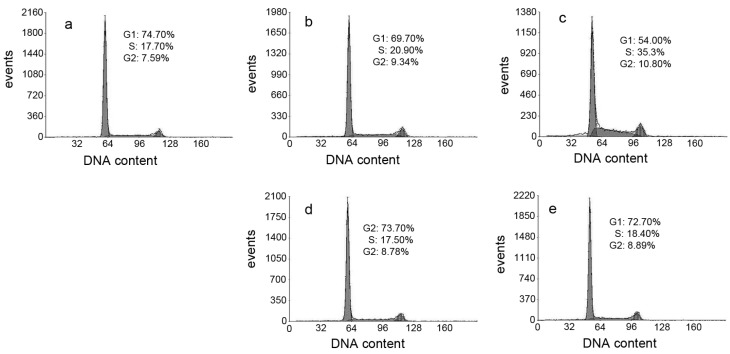
Cell cycle distribution of HepG2 cells following treatment with various concentrations of DpdtpA and its Cu complex: (**a**) Control; (**b**) 1.56 μM DpdtpA; (**c**) 3.12 μM DpdtpA; (**d**) 1.56 μM DpdtpA–Cu; and (**e**) 3.12 μM DpdtpA–Cu.

ROS induce cell cycle delay at the G1/S boundary [26]. Therefore, we evaluated the effect of DpdtpA and its copper complex on the cell cycle distribution using propidium iodide staining and flow cytometry. As shown in Figure 7, DpdtpA caused an accumulation of the HepG2 cells in the *S*-phase, and the percentage of the cells at the *S*-phase significantly increased from 17.70% to 20.90% or 35.3% upon exposure of the cells to DpdtpA for 24 h (Figure 7b,c). However, it is interesting that treatment with DpdtpA–Cu complex (Figure 7d,e) did not alter the cell cycle of the host cell, indicating that there was a difference in the induced cell cycle arrest.

### 2.7. The Change in Lysosomal (Autophagosome) Membrane Permeability (LMP) in Cells upon Exposure to DpdtpA and Its Copper Complex

**Figure 8:**
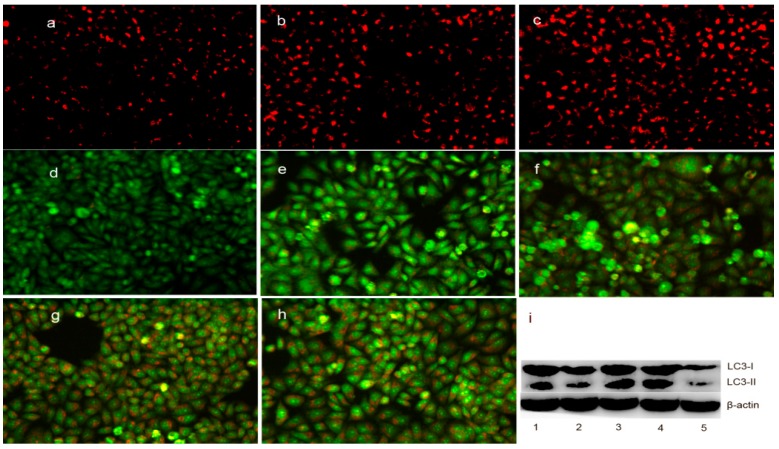
DpdtpA and its copper complex induced autophagy (or changes in LMP) in HepG2 cells: (**a**) LysoTracker Red-stained HepG2 cells (control); (**b**) 0.78 μM DpdtpA; (**c**) 0.78 μM DpdtpA–Cu; (**d**) control; (**e**) 0.78 μM DpdtpA; (**f**) 1.56 μM DpdtpA; (**g**) 0.78 μM DpdtpA–Cu; (**h**) 1.5 μM DpdtpA–Cu; and (**i**) Western blotting: 1, 0.78 μM DpdtpA; 2, 1.56 μM DpdtpA; 3, 0.78 μM DpdtpA–Cu; 4, 1.5 μM DpdtpA–Cu; and 5, control.

As mentioned above, the upregulation of bax may also be involved in the translocation from the cytosol to the lysosomal membrane, which can regulate the lysosomal membrane integrity [27]. To test this hypothesis, LysoTracker Red, which can accumulate within the lysosomes, was employed to assess the LMP [28]. As shown in Figure 8a–c, the red fluorescence density in HepG2 cells significantly increased in the DpdtpA- and DpdtpA–Cu-treated groups compared to that in the control, indicating that LMP was altered. Furthermore, the changes in LMP may be a response to autophagy; thus, the formation of autophagosomes was measured by acridine orange staining. As shown in Figure 8d–h, the red granular fluorescence in the acidic vacuoles was observed in the treated groups and follows a concentration-dependent pattern, which implied that autophagy occurred. For further determination of the involvement of autophagy, LC3 (microtubule-associated protein light chain 3), an autophagosome marker, was measured by Western blotting analysis. As expected, an increase in the cleaved LC3-II and a decrease in LC3-I were observed in the treated groups compared to that in the control, indicating that autophagy was involved in cellular death after exposure to the test compounds (Figure 8i). The results demonstrated the DpdtpA and its copper complex could induce autophagy in the same manner. However, it was clear that the stronger autophagy induction and destruction of LMP by DpdtpA–Cu did not correlate with its antiproliferative activity.

## 3. Discussion

Although many methods have been developed for cancer therapy and diagnosis, cancer treatment is still a challenge. This can be attributable to the lack of both a diagnostic method that can identify the early phase of the disease and a highly selective drug that can target the cancer cells. However, recent studies revealed that the tumor microenvironment plays a critical role in the cancer growth, metastasis, and treatment responses [29]. Targeting the tumor microenvironment was recently proposed as a strategy for cancer therapy [4]. The tumor microenvironment consists of tumor cells, ECM, molecules in the ECM, and other cells, such as fibroblasts and immune cells [2]. The molecules in the ECM contain many metalloenzymes and cytokines, which can be used as drug targets. Thus, the involvement of metal ions in the ECM in homeostasis can be used as a target for chelating therapy. In this strategy, the decrease of chelator in cytotoxicity upon binding to the metal may be a good sign. Pyrrolidine dithiocarbamate (PDTC), one of the members of the dithiocarbamate family, displays a dual action, including an antioxidant and radical scavenging activity [30,31,32] and apoptosis induction capability. It is a well accepted concept in antitumor drug design that excess ROS can cause oxidative damage of proteins and nucleic acids, and accordingly can result in cell death. Our data showed that DpdtpA exhibited the ability to generate ROS both in vitro and in vivo, which correlated with its cytotoxicity. ROS production induced by DpdtpA may be due to Fenton-like reaction (redox-active DpdtpA-Fe^2+^) and catalase inhibition (data not shown). We speculated that DpdtpA could chelate ferrous iron from the iron labile pool, and form the redox-active DpdtpA-Fe^2+^ species to produce excess ROS that cause lipid oxidation and DNA fragmentation [33]. To determine the correlation between ROS production and DNA cleavage, the comet assay was also conducted. It showed that DpdtpA induced cellular DNA breakage in a concentration-dependent manner. Further evidence from Western blotting analysis supported the hypothesis that apoptosis was involved because bax and caspases were upregulated, whereas bcl-2 was downregulated after exposure of the HepG2 cells to DpdtpA. These changes are frequently observed together in the cells subjected to anticancer drug treatment [34]. In addition, DpdtpA can induce cell cycle arrest at the S phase as reported [35,36]. To gain insight into the detailed mechanism, cyclin D1, an S phase cell cycle gatekeeper protein, was measured. Cyclin D1 was downregulated (Figure 7), which implied that suppressed cyclin D1 favored the entry into the S phase because it inhibits DNA synthesis by virtue of its binding to the regulator of DNA synthesis [37].

Copper is an essential element for cell growth. Many drugs, used in clinical practice, have metal-chelating ability and display cytotoxicity. In vitro copper complexes generally show an enhanced antiproliferative activity [24,25,38,39] and an antagonistic effect was rarely reported. Dithiocarbamates are known to act as inhibitors of the canonical NF-κB pathway since 1992 [21], whereas dithiocarbamate copper complexes exhibit a distinct biological activity compared to dithiocarbamates [40]. It has been demonstrated that proteasome inhibition by dithiocarbamate copper complexes contributes to their cytotoxicity [41,42,43,44]; however, these studies reported that dithiocarbamates showed an enhanced cytotoxicity upon chelation with copper. In the present study, we showed that copper ion dramatically attenuated the antiproliferative activity of DpdtpA against the given cell lines, an effect which has not been reported for any dithiocarbamate derivative (Figure 1). The “antagonistic effect” was confirmed to be due to the formation of new species (copper chelate) by spectral titration of DpdtpA against copper ion solution. The new species was formed at a ratio of DpdtpA:Cu = 2. The possible structure of the new species was proposed based on the IR and other spectral data. Second, we observed a paradoxical relation between the cellular ROS level and cytotoxicity. DpdtpA–Cu exhibited a stronger ROS-inducing ability in vitro and in vivo compared to DpdtpA; however, the changes in the expression of apoptotic proteins and DNA fragmentation were not evident, which violated the currently accepted concept in ROS-based cancer therapy. Similarly, Zhu et al. recently reported that no dose–response relationship was evident between the cellular ROS level and cytotoxicity [45]. It was noted that although DpdtpA–Cu and DpdtpA had similar effects regarding apoptosis induction, the difference in the cell cycle delay was distinct. DpdtpA caused cell cycle arrest at the S phase, whereas DpdtpA–Cu did not exhibit this effect, which may be an important contributor to its weaker antiproliferative activity.

Autophagy functions to degrade the damaged proteins and/or organelles and to recycle the materials to maintain the quality of cellular components [46]. The formation of acidic vesicular organelles (AVOs) is a characteristic marker of the autophagy process [47]. ROS-triggered autophagy has been widely realized [48]. In view of the higher ROS level reported in vivo, we speculated that the antiproliferative activity of DpdtpA or its copper complex might be due to autophagy. As expected, the granular red fluorescence in AVOs was observed after treatment of HepG2 cells with the test compounds (Figure 7d–h), suggesting that autophagy was involved. Further supporting evidence was from the immunofluorescence detection of LC3, a marker of autophagy. LC3-I levels decreased, whereas LC3-II levels increased (Figure 8i). DpdtpA–Cu exhibited a marked ability to form autophagosomes and lysosomes than DpdtpA, which correlated with ROS production, but not with its antiproliferative activity. The underlying reason was not clear.

In conclusion, DpdtpA exhibited a significant antiproliferative activity against hepatocellular carcinoma cells via ROS-meditated apoptosis, autophagy, and cell cycle arrest. However, the addition of copper dramatically decreased its antiproliferative activity due to the formation of a chelate. Although ROS production due to DpdtpA–Cu cytotoxicity was obvious, the correlation between ROS production and cytotoxicity was not established. This was similar to the results of recent reports. The difference between DpdtpA and DpdtpA–Cu regarding the antiproliferative activity could be explained based on the observation that the former had a distinct ability to disturb the cell cycle, but the latter did not. Additionally, the attenuated antiproliferative activity of DpdtpA upon copper chelation is considered as an advantage for animal studies to target the tumor microenvironment. However, the paradoxical relationship between ROS production and cytotoxicity of DpdtpA–Cu requires further investigation.

## 4. Materials and Methods

### 4.1. General Information

MTT (3-(4,5-dimethylthiazol-2-yl)-2,5-diphenyltetrazolium bromide), ethidium bromide (EB), di-2-pyridylketone, RPMI-1640, and other chemicals were purchased from Sigma-Aldrich (Shanghai, China). LC3 antibody was obtained from Proteintech Group (Wuhan, China). Antibodies against cyclin D1, caspase 3, β-actin, Bax, and Bcl-2 were purchased from Boster (Wuhan, China).

### 4.2. Preparation of Di-2-pyridylhydrazone Dithiocarbamate S-propionic Acid (DpdtpA)

DpdtpA (chemical name generated by ACDLabs: 3-[({2-[di(pyridin-2-yl)methylidene]hydrazinyl} carbonothioyl)sulfanyl]propanoic acid) was prepared via a three-step reaction as indicated in Figure 1. First, the hydrazine dithiocarbamate was synthesized by the reaction of equimolar carbon disulfide (1 mmol) with hydrazine (1 mmol) in KOH-containing ethanol (10 mL) on an ice bath for 1 h [49]. Then, the reaction mixture, without further separation, was mixed with an equimolar amount of di-2-pyridylketone (1 mmol). The resulting mixture was refluxed for 1 h. After cooling, the red-brown solid was filtered and washed with cold ethanol. Thin-layer chromatography (TLC) showed one spot (mobile phase:ethyl acetate/petroleum ether = 3:1). The unpurified product showed a yield of 80% and a melting point (mp) of 140.5 °C. The di-2-pyridylhydrazone dithiocarbamate was characterized by: ^1^H-NMR (Bruker, DMSO-*d*_6_, ppm): 13.35 (s, NH), 8.85 (d, H, *J* = 4 Hz), 8.63 (d, H, *J* = 4 Hz), 8.03 (m, 2H, *J* = 8 Hz), 7.95 (d, H, *J* = 8 Hz), 7.63 (dd, H, *J* = 4 Hz), 7.59 (d, H, *J* = 8 Hz), 7.54 (dd, H, *J* = 4 Hz); IR (PerkinElmer, Life and Analytical Sciences, Beaconsfield, UK; KBr, cm^−1^): 3430, 1624, 1587, 1519, 1461, 1430, 1217, 1187, 1133, 1051, 1012, 992, 800, 753, 731, 712, 648, 618, and 590; ESI-MS (microTOF-Q III, Bruker, *m*/*z*): 350.9540 (M − H + 2K, calcd: 350.9525).

Next, the red-brown solid (di-2-pyridylhydrazone dithiocarbamate, 1 mmol) was dissolved in absolute ethanol (5 mL), and reacted with 3-bromopropionic acid at room temperature for 1 h. Then, the yellow solid was filtered and washed with ethanol. TLC tracing (ethyl acetate/petroleum ether = 3:1) showed that the reaction is complete. The yield was 90% and mp was 155 °C. ^1^H-NMR (DMSO-*d*_6_, ppm): 15.0 (s, NH), 8.85 (d, H, *J* = 4 Hz), 8.63 (d, H, *J* = 4 Hz), 8.03 (m, 2H, *J* = 8 Hz), 7.95 (d, H, *J* = 8 Hz), 7.63 (dd, H, *J* = 4 Hz), 7.59 (d, H, *J* = 8 Hz), 7.54 (dd, H, *J* = 4 Hz), 3.43 (tri, 2H, *J* = 8 Hz), 2.71 (tri, H, *J* = 8 Hz). IR (KBr, cm^−1^): 3404, 1701, 1587, 1458, 1356, 1329, 1288, 1234, 1207, 1133, 1061, 1034, 1014, 803, 753, 701, 654, and 593. ESI-MS (*m*/*z*): 385.0203 (M + K, calcd: 385.01954).

DpdtpA copper complex (DpdtpA–Cu) was prepared by two methods: (1) During the cytotoxicity assay: DpdtpA–Cu was formed by mixing 100 mM DpdtpA in dimethylformamide (DMSO, 100 µL) with 1 M copper chloride in water (5 µL); (2) DpdtpA–Cu was prepared by mixing 0.1 mmol DpdtpA in ethanol with 0.05 mmol copper chloride solution with stirring, the resulting solution was further stirred overnight at room temperature (protected from light), then after filtration, the powder was dried in a vacuum desiccator. The yield was 70% and mp was 198.4 °C. IR (cm^−1^): 3404, 1731, 1708, 1593, 1476, 1438, 1407, 1385, 1327, 1185, 1118, 1095 and 996. IR and HPLC analyses are shown in Figures S3 and S4). The comparison between the IR of DpdtpA and DpdtpA–Cu is shown in Figure S5. Accordingly, the possible coordination structure of DpdtpA–Cu is tentatively proposed in Figure S6.

### 4.3. Cytotoxicity Assay (MTT Assay)

DpdtpA (10 mM) in 80% DMSO was diluted to the required concentration with the culture medium. The copper complex was prepared by mixing DpdtpA with CuCl_2_ (at high concentration) based on a 1:2 molar ratio and diluted to the required concentration with water. The MTT assay was conducted as previously described [25]. Briefly, 5 × 10^3^/mL Bel-7402 cells (or HepG2 cells) in the exponential phase were seeded equally into a 96-well plate and various amounts of DpdtpA (or its copper complex) were added after the cells adhered. After 48-h incubation at 37 °C in a humidified atmosphere containing 5% CO_2_, 10 µL of MTT solution (5 mg/mL) was added to each well followed by additional 4-h incubation. The cell culture medium was removed and 100 μL of DMSO was added to each well to dissolve the formazan crystals. The measurement of the absorbance of the solution, which is related to the number of living cells, was performed using a microplate reader (MK3, Thermo Scientific) at 570 nm. Growth inhibition percentage was defined as percentage of absorbance inhibition within appropriate absorbance in each cell line. The assay was performed in triplet. A morphological study was conducted using an inverted microscope (Shanghai Batuo Instrument Co., Ltd., Shanghai, China), and the photographs of HepG2 cells exposed to DpdtpA (2.5 or 5.0 µM DpdtpA or DpdtpA–Cu) for 16 or 48 h were recorded (objective lens size: 10 × 20).

### 4.4. Determination of the Molar Ratio of DpdtpA to Copper(II) Involved in the Formation of the Complex

The stoichiometry of the reaction between DpdtpA and copper chloride was determined as previously described [25]. Briefly, solutions of 1 mM DpdtpA in 50% DMSO and 1 mM CuCl_2_ in water were prepared. Next, 0.04 mL of the CuCl_2_ solution was added to a series of 5 mL-volumetric flasks, and then a different volume of DpdtpA (10, 20, 30, 40, 50, 60, 70, 80, 85, and 90 µL) was added to each flask. Finally, 50 mM Tris-HCl buffer (pH 7.4) was added to total 5 mL. After mixing and equilibrium, the UV spectra were recorded on a Shimadzu-UV-2450 spectrophotometer (Shimadzu Co., Ltd., Suzhou, China).

### 4.5. ROS Detection in Vitro and in Vivo

Assessment of ROS production was conducted as previously reported [25]. Briefly, H_2_DCF-DA was first converted to DCF by NaOH, and the neutralized hydrolysate was used for the in vitro assay. The reaction system contained either a single reagent or multiple components in 50 mM sodium phosphate buffer (pH 7.4) with a total volume of 4 mL (i.e., 0.4 μM DCF, 6.25 μM NH_4_FeSO_4_ (or CuCl_2_ or DpdtpA) and 200 μM H_2_O_2_ (1 mM) for Fenton reaction). The fluorescence was detected by a FC-960 spectrofluorometer (excitation wavelength at 488 nm and emission wavelength at 525 nm, Shanghai Lengguang Technology Co., Ltd., Shanghai, China) within a 10-min time course at room temperature.

The intracellular ROS assay was carried out in accordance with the company’s recommendations (Beyotime Biotechnology, Beijing, China). Approximately 10^6^ HepG2 cells were used in the assay. After washing with phosphate-buffered saline (PBS), the cells were re-suspended in H_2_DCF-DA-containing serum-free culture medium and incubated for 30 min. Next, the stained cells were washed with serum-free culture medium. Then, 100 μL of the cell culture was transferred to individual PCR tubes and the test compound (or positive control) was added. After 1-h incubation, the cell suspension was used directly for ROS detection by a FC-960 spectrofluorometer (excitation wavelength at 488 nm and emission wavelength at 525 nm, Shanghai Lengguang Technology Co., Ltd., Shanghai, China).

### 4.6. Comet Assay

DNA fragmentation was determined by the comet assay as previously described [24]. HepG2 cells were treated with or without the investigated compounds (0.78 and 1.56 µM) and incubated for 24 h in a humidified atmosphere of 5% CO_2_. The harvested cells were then embedded in 0.5% low-melting point agarose at 10^4^ cells/mL. Forty microliters of this cellular suspension was spread onto duplicate frosted slides that were covered with 1% normal-melting point agarose. Upon solidification, the slides were placed in a lysis buffer (2.5 M NaCl, 0.1 M ethylenediaminetetraacetic acid (EDTA), 0.01 M Tris-HCl, 1% Triton X-100, 10% DMSO, pH 10) for 1 h at 4 °C. After lysis, the slides were transferred to an alkaline buffer for 40 min (0.001 M EDTA, 0.3 M NaOH, pH > 13) to allow the DNA to unwind before migration at 0.66 V/cm and 300 mA for 30 min. All of these steps were performed in the dark. After neutralization in 0.4 M Tris-HCl, pH 7.4, the slides were stored at 4 °C until analysis within the following 24 h. Before analysis, the slides were stained with ethidium bromide (20 μg/mL) and covered with coverslips. Images were captured using a fluorescent microscope (Shanghai Batuo Instrument Co. Ltd., Shanghai, China).

### 4.7. Western Blotting Analysis

Briefly, 1 × 10^7^ HepG2 cells treated with or without DpdtpA or DpdtpA–Cu were scraped off in a lysis buffer (50 mM Tris-HCl, pH 8.0, 150 mM NaCl, 1.0% NP-40, 10% glycerol, and protease inhibitors) and subjected to sonication followed by spinning down by centrifugation at 14,000× *g*. The clear supernatant was stored at −80 °C. The protein concentration was determined using the colorimetric Bio-Rad DC protein assay on a microplate reader, MK3, at 570 nm. Proteins (50 μg) were separated on a 13% sodium dodecyl sulfate-polyacrylamide gel at 200 V for 1 h. Then, the separated proteins were subsequently transferred onto a polyvinylidene difluoride (PVDF) membrane at 60 V for 1 h. The membrane was washed three times with tris-buffered saline (TBS), and was then blocked for 2 h in TBS containing 0.1% Tween-20 and 5% skimmed milk. The membrane was incubated at 4 °C overnight with the primary monoclonal antibody used at a dilution of 1:300 in TBS plus 0.1% Tween-20 (TBST). The membrane was washed several times with TBST and was subsequently incubated with horseradish peroxidase (HRP)-conjugated secondary antibody (1:2000 in TBST) for 1 h at room temperature. After another wash of the membrane with TBST, the protein bands were detected using a super-sensitive ECL solution (Boster Biological Technology Co., Ltd., Wuhan, China), and visualized using an Amersham imager 600 (GE Healthcare Life Sciences, Fairfield, CT, USA).

### 4.8. Cell Cycle Analysis

HepG2 cells (1 × 10^5^) were seeded in a 6-well plate and incubated for 24 h at 37 °C (5% CO_2_). The medium was replaced with fresh medium supplemented or not (control) with the test compounds (1.5 and 3.12 μM). After 24-h incubation, the cells were harvested with trypsin, followed by washing with PBS, and were fixed in 70% ethanol and stored at −20 °C. The cellular nuclear DNA was stained using propidium iodide (PI). Briefly, after removing the 70% ethanol, the cells were washed with PBS and then suspended in 0.5 mL PBS containing 50 g/mL PI and 100 μg/mL RNase. The cell suspension was incubated at 37 °C for 30 min. DNA flow cytometry was performed in duplicate using a FACSCalibur flow cytometer (Becton-Dickinson, San Jose, CA, USA). For each sample, 10,000 events were collected, and the fluorescence signal intensity was recorded and analyzed by CellQuest and ModiFit (Becton-Dickinson).

### 4.9. DpdtpA and Its Copper Complex Induced Autophagy

Cells were seeded into a 24-well flask and treated as previously described for the cell viability assay. The cells were treated with different concentrations of the test compounds (0.78 and 1.56 μM DpdtpA or DpdtpA–Cu) for 24 h. For detection of the acidic cellular compartment, acridine orange (Genview, Jacksonville, FL, USA) was used, which emits bright red fluorescence in the acidic vesicles, whereas it emits green fluorescence in the cytoplasm and nucleus. After treatment of the cells with the test compounds, acridine orange was added at a final concentration of 1 μg/mL (the concentration of LysoTracker Red (Beyotime Biotechnology, Shanghai, China), as recommended) for 15 min. After PBS washing, the fluorescence micrographs were captured using an inverted fluorescence microscope.

## Supplementary Materials

The following are available online at http://www.mdpi.com/1420-3049/21/8/1088/s1.

## Abbreviations

The following abbreviations are used in this manuscript:

MTT	3-(4,5-dimethylthiazole-2-yl)-2,5-diphenyl tetrazoliumbromide
DpdtpA	Di-2-pyridylhydrazone dithiocarbamate *S*-propionic acid
DpdtpA–Cu	Copper complex of di-2-pyridylhydrazone dithiocarbamate *S*-propionic acid
ROS	Reactive oxygen species
ECM	Extracellular matrix
NMR	Nuclear magnetic resonance
IR	Infrared
