# Supplementary material for: Copper Ion Attenuated the Antiproliferative Activity of Di-2-pyridylhydrazone Dithiocarbamate Derivative; However, There Was a Lack of Correlation between ROS Generation and Antiproliferative Activity
Source: Molecules. 2016 Aug 20;21(8):1088. doi: 10.3390/molecules21081088 (PMC6273760; doi:10.3390/molecules21081088)
Supplement: Supplementary file 1 [file molecules-21-01088-s001.pdf]

Supplementary Materials: Copper Ion Attenuated the Antiproliferative Activity of Di-2-Pyridylhydrazone Dithiocarbamate Derivative; However, There Was a Lack of Correlation between ROS Generation and Antiproliferative Activity

Tingting Wang, Yun Fu, Tengfei Huang, Youxun Liu, Meihao Wu, Yanbin Yuan, Shaoshan Li and Changzheng Li

1. Purity of DpdtpA Was Determined by HPLC and NMR

1.1. HPLC (Shimadzu Corpation, Kyoto, Japan) Isolation(Gradient: 20%–50% Solven B within 10 min, Following Increased to 100% in 10 min, and Decreased to 20% in 2 min. and Keep Same Percent to 30 min). Solvent A: Water Plus 0.1% TFA; Solvent B: Acetonitrile Plus 0.1% TFA)

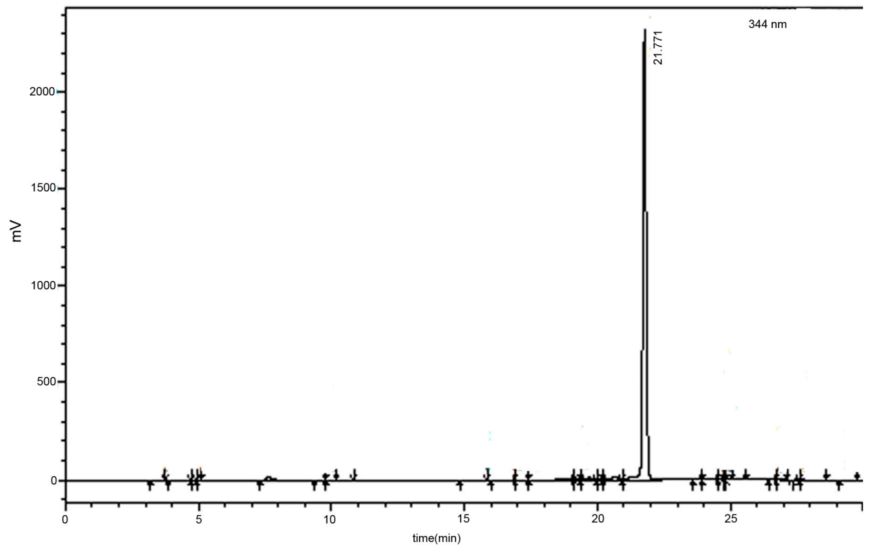

Figure S1. HPLC of DpdtpA

1.2. ^{1}HNMR Spectrum of DpdtpA

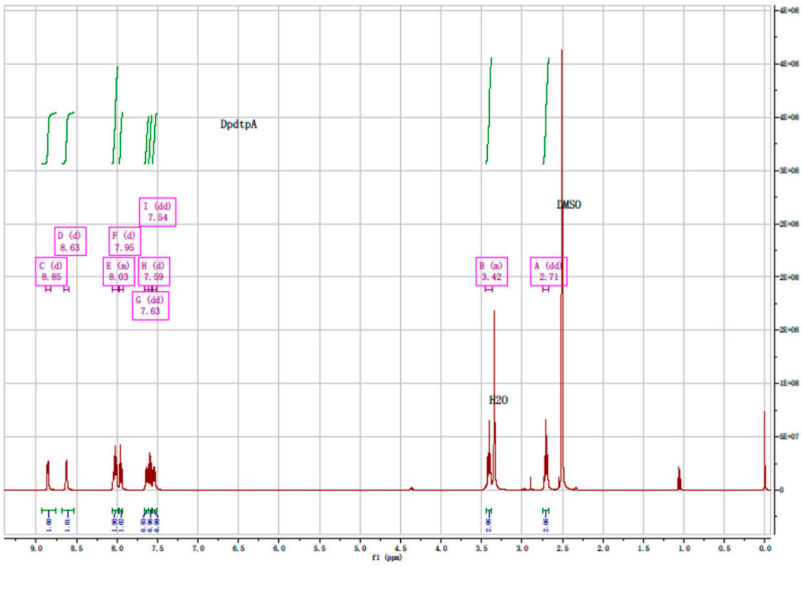

Figure S2. HNMR spectrum of DpdtpA.

2. Purity of DpdtpA-Cu

2.1. HPLC(Shimadzu Corpation, Kyoto, Japan) Isolation(Gradient: 20%–50% Solven B within 10 min, Following Increased to 100% in 10 min, and Decreased to 20% in 2 min. and Keep Same Percent to 30 min). Solvent A: Water Plus 0.1% TFA; Solvent B: Acetonitrile Plus 0.1% TFA)

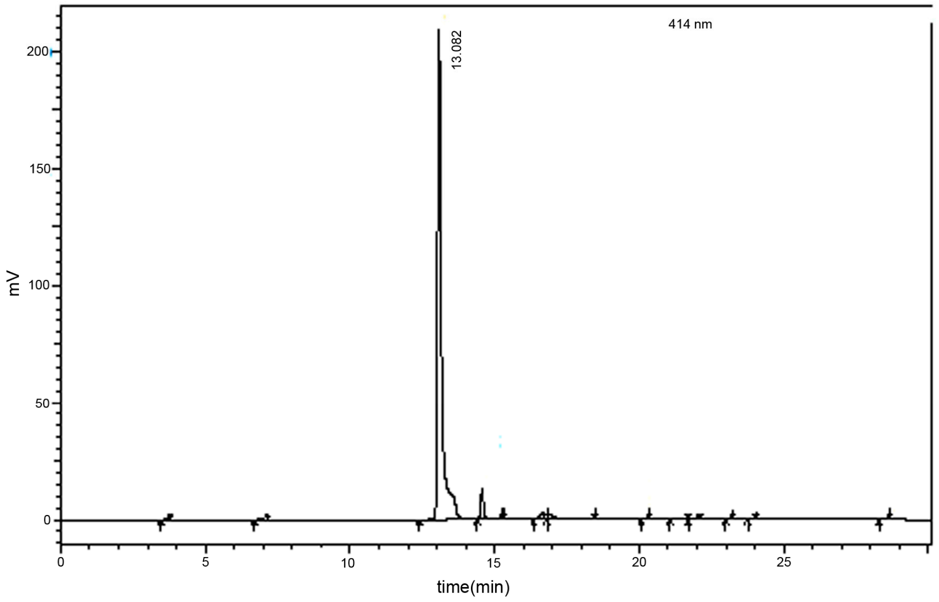

Figure S3. HPLC of dpdtpA-Cu.

2.2. IR Spectrum of DpdtpA-Cu

Based on literature [1,2], 3273–3493 cm^{-1} asigned to υ_{NH} of amide, 1457 cm^{-1} assigned to $\upsilon_{C\text{-}N}$ stretching frequency for the thioureide group in the DpdtpA, and 1230 cm^{-1} were assigned to C=S bond, but in the DpdtpA-Cu, the peaks were disappeared, indicating enolization occurred [1,2].

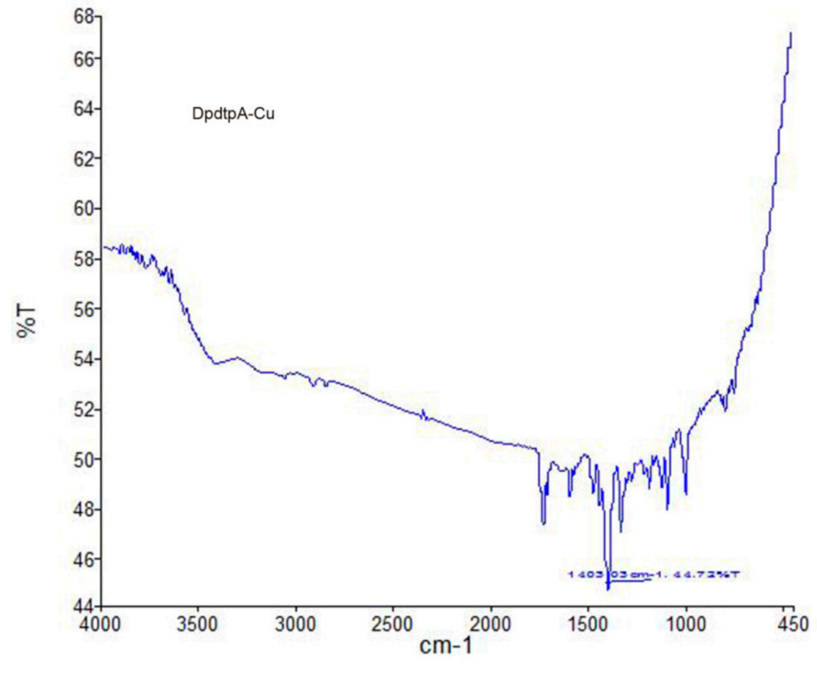

Figure S4. IR spectrum of DpdtpA-Cu.

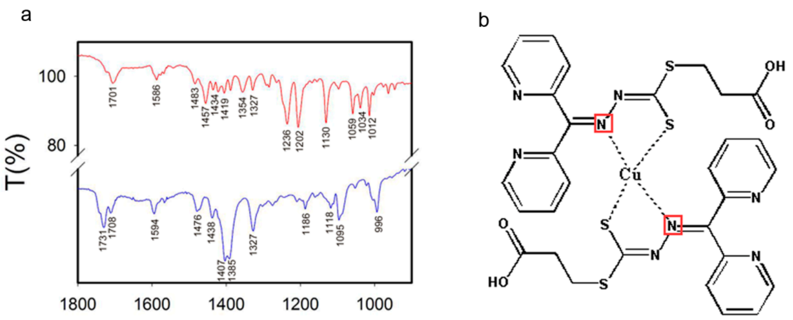

Figure S5. (**a**) IR comparison between DpdtpA (**red** line) and DpdtpA-Cu (**blue** line); and (**b**) coordination structure of DpdtpA-Cu proposed tentatively.

References

1. SaLlJk, B.N.; Özkay, Y.; Özkay, Ü.D.; Gençer, H.K. Synthesis and biological evaluation of some novel dithiocarbamate derivatives. *J. Chem.* **2014**, doi:10.1155/2014/387309.
2. Nabipour, H. Synthesis of a new dithiocarbamate cobalt complex and its nanoparticles with the study of their biological properties. *Int. J. Nano Dim.* **2011**, *1*, 225–232.
